# Hyperglycemic adverse events following antipsychotic drug administration in spontaneous adverse event reports

**DOI:** 10.1186/s40780-015-0015-6

**Published:** 2015-04-16

**Authors:** Yamato Kato, Ryogo Umetsu, Junko Abe, Natsumi Ueda, Yoko Nakayama, Yasutomi Kinosada, Mitsuhiro Nakamura

**Affiliations:** Laboratory of Drug Informatics, Gifu Pharmaceutical University, 1-25-4, Daigaku-Nishi, Gifu, 501-1196 Japan; Medical Database Co., LTD, 3-11-10, Higashi, Sibuya Ward, Tokyo, 150-0011 Japan; Department of Biomedical Informatics, Gifu University Graduate School of Medicine, 1-1 Yanagido, Gifu, 501-1196 Japan

**Keywords:** Antipsychotic drugs, Hyperglycemic adverse events, Adverse event reporting system, Antipsychotic polypharmacy, Antipsychotic monotherapy

## Abstract

**Background:**

Antipsychotics are potent dopamine antagonists used to treat schizophrenia and bipolar disorder. The aim of this study was to evaluate the relationship between antipsychotic drugs and adverse hyperglycemic events using the FDA Adverse Event Reporting System (FAERS) database. In particular, we focused on adverse hyperglycemic events associated with atypical antipsychotic use, which are major concerns.

**Findings:**

We analyzed reports of adverse hyperglycemic events associated with 26 antipsychotic drugs in the FAERS database from January 2004 to March 2013. The Standardized Medical Dictionary for Regulatory Activities Queries (SMQ) preferred terms (PTs) was used to identify adverse hyperglycemic events. The number of adverse hyperglycemic reports for the top eight antipsychotic drugs, quetiapine, olanzapine, risperidone, aripiprazole, haloperidol, clozapine, prochlorperazine, and chlorpromazine was 12,471 (28.9%), 8,423 (37.9%), 5,968 (27.0%), 4,045 (23.7%), 3,445 (31.5%), 2,614 (14.3%), 1,800 (19.8%), and 1,003 (35.7%), respectively. The reporting ratio increased with co-administration of multiple antipsychotic drugs. For example, adverse hyperglycemic events represented 21.6% of reports for quetiapine monotherapy, 39.9% for two-drug polypharmacy, and 66.3% for three-drug polypharmacy.

**Conclusion:**

Antipsychotic drug polypharmacy may influence signal strength, and may be associated with hyperglycemia. After considering the causality restraints of the current analysis, further robust epidemiological studies are recommended.

## Findings

### Background

Antipsychotics are potent dopamine antagonists used to treat schizophrenia and bipolar disorder [1]. Antipsychotics are categorized as first-generation antipsychotics (typical) and second-generation antipsychotics (atypical). Several studies have reported abnormal glucose metabolism during antipsychotic drug treatment [2-4]. In 2002, diabetic ketoacidosis and coma were reported after olanzapine and quetiapine treatment in Japan [5]. Furthermore, the Food and Drug Administration (FDA) asked manufacturers of atypical antipsychotic (AAP) drugs to add a warning to drug labels regarding the increased risk of hyperglycemia and diabetes in 2004 [6]. Thus, hyperglycemia due to antipsychotic drug administration is a serious clinical problem.

According to clinical practice guidelines, AAPs should be used as the first and second line of treatment following the first schizophrenic episode [7-10]. However, treatment resistance and poor efficacy continue to be a significant clinical problem [2,11,12]. Since antipsychotic polypharmacy is suggested after failure of antipsychotic monotherapy [7,9,10], multiple antipsychotic drugs have been frequently prescribed [2,11,13]. A case-control study indicated that the administration of multiple antipsychotics increases the risk of diabetes mellitus when using AAPs [1]. Several studies also demonstrated the effect of antipsychotic polypharmacy on the adverse events; however, the effect of antipsychotic polypharmacy on hyperglycemia remains unclear [11-14].

The FDA Adverse Event Reporting System (FAERS) is a spontaneous reporting system for adverse events. It is the largest and best-known database worldwide, and reflects the realities of clinical practice. Therefore, the FAERS database is one of the primary tools used in pharmacovigilance. The aim of this study was to evaluate the relationship between antipsychotic drugs and adverse hyperglycemic events using the FAERS database. To our knowledge, this study is the first to evaluate the effect of antipsychotic polypharmacy on adverse hyperglycemic events using the FAERS database.

### Methods

Data from the FAERS database from January 2004 to March 2013 were obtained from the FDA website. The FAERS database structure complies with the International Conference on Harmonization (ICH) E2B. We analyzed 26 antipsychotic drugs associated with hyperglycemia (Table 1). Since drug names in the FAERS database are registered arbitrarily, DrugBank, a reliable drug database, was utilized as a dictionary for the batch conversion and compilation of drug names (Table 2). We followed the FDA’s recommendation to adopt the most recent case number in order to identify duplicate reports from the same patient and excluded these from analysis.

**Table 1:** **Characteristics of antipsychotics in the FDA adverse event reporting system database**

**Drugs**	**Total**	**Cases** ^*^	**Reporting ratio (%)**	**ROR (95%CI)**	
*Atypical*	96841	21151	21.8	2.5	(2.4-2.5)
Aripiprazole	17093	4045	23.7	2.6	(2.5-2.7)
Clozapine	18217	2614	14.3	1.4	(1.3-1.5)
Olanzapine	22200	8423	37.9	5.3	(5.1-5.4)
Quetiapine	43169	12471	28.9	3.5	(3.4-3.6)
Perospirone	83	26	31.3	3.8	(2.4-6.1)
Risperidone	22121	5968	27.0	3.1	(3.0-3.2)
Zotepine	134	31	23.1	2.5	(1.7-3.8)
*Typical*	19569	3948	20.2	2.1	(2.1-2.2)
Bromperidol	48	11	22.9	2.5	(1.3-4.9)
Chlorpromazine	2812	1003	35.7	4.6	(4.3-5.0)
Fluphenazine	923	234	25.4	2.8	(2.4-3.3)
Haloperidol	10922	3445	31.5	3.9	(3.7-4.0)
Levomepromazine	799	166	20.8	2.2	(1.8-2.6)
Moperone	0	0	-	-	
Nemonapride	4	1	25.0	2.8	(0.3-26.8)
Perphenazine	911	341	37.4	5.0	(4.4-5.7)
Pimozide	246	65	26.4	3.0	(2.3-4.0)
Pipamperone	207	26	12.6	1.2	(0.8-1.8)
Prochlorperazine	9103	1800	19.8	2.1	(2.0-2.2)
Propericiazine	190	45	23.7	2.6	(1.9-3.6)
Spiperone	1	0	-	-	
Sulpiride	1809	331	18.3	1.9	(1.7-2.1)
Sultopride	97	11	11.3	1.1	(0.6-2.0)
Thioridazine	574	160	27.9	3.2	(2.7-3.9)
Tiapride	336	81	24.1	2.7	(2.1-3.4)
Timiperone	15	4	26.7	3.0	(1.0-9.5)
Trifluoperazine	619	274	44.3	6.6	(5.7-7.8)

*With adverse events of interest.

**Table 2:** **Generic names and brand names of antipsychotics in the DrugBank**

	**Generic name**	**Brand name**
*Atypical*		
	Aripiprazole	Abilify, Aripiprazole
	Clozapine	Clozapin, Clozapine, Clozaril, Fazaclo odt, Leponex
	Olanzapine	Olansek, Olanzapine, Symbyax, Zydis, Zyprexa, Zyprexa intramuscular, Zyprexa zydis
	Quetiapine	Quetiapine, Quetiapine fumarate, Seroquel, Seroquel xr
	Risperdone	Risperdal, Risperdal consta, Risperdal m-tab, Risperdone, Risperidona, Risperidone, Risperidonum, Risperin, Rispolept
*Typical*		
	Chlorpromazine	Chlorpromanyl, Chlorpromazine, Largactil, Thorazine
	Haloperidole	Aloperidin, Aloperidol, Aloperidolo, Apo-haloperidol, Haldol, Haldol la, Haldol solutab, Haloperidol, Haloperidol decanoate, Haloperidol lactate, Halopidol, Halosten, Keselan, Linton, Novo-peridol, Peridol, Serenace
	Prochloroperazine	Buccastem, Chlorperazine, Combid, Compazine, Compro, Emetiral, Novamin, Pasotomin, Prochloroperazine, Prochlorpemazine, Prochlorperazin, Prochlorperazine, Prochlorperazine edisylate, Prochlorperazine maleate, Prochlorpromazine, Procloperazine, Proclorperazine, Stemetil, Stemzine, Vertigon

Adverse events in the FAERS database are coded according to the terminology preferred by the Medical Dictionary for Regulatory Activities (MedDRA). The Standardized MedDRA Queries (SMQ) index is widely accepted and utilized in the analysis of the FAERS database [15]. We utilized the SMQ for *hyperglycemia/new onset diabetes mellitus* events (SMQ code: 20000041). We selected 93 Preferred Terms (PTs), which are summarized in Table 3.

**Table 3:** **Preferred terms associated with adverse hyperglycemia in the Standardized MedDRA Queries (SMQ; 20000041)**

**Preferred terms**	**Code**	**Total**	**Atypical**		**Typical**	
			**Cases** ^*^	**Reporting ratio (%)**	**Cases** ^*^	**Reporting ratio (%)**
Total		241478	21151	8.8	3948	1.6
Abnormal loss of weight	10000159	532	28	5.3	9	1.7
Abnormal weight gain	10000188	134	33	24.6	0	0
Acidosis	10000486	1956	102	5.2	44	2.2
Altered state of consciousness	10001854	3306	303	9.2	111	3.4
Anti-GAD antibody positive	10059728	23	2	8.7	0	0
Anti-insulin antibody increased	10053815	51	0	0	0	0
Anti-insulin antibody positive	10053814	115	0	0	0	0
Anti-insulin receptor antibody increased	10068226	0	0	0	0	0
Anti-insulin receptor antibody positive	10068225	3	0	0	0	0
Anti-islet cell antibody positive	10049439	4	1	25	0	0
Blood 1,5-anhydroglucitol decreased	10065367	0	0	0	0	0
Blood cholesterol increased	10005425	10887	1648	15.1	63	0.6
Blood glucose abnormal	10005554	1547	116	7.5	12	0.8
Blood glucose fluctuation	10049803	2267	76	3.4	6	0.3
Blood glucose increased	10005557	35838	1398	3.9	241	0.7
Blood insulin abnormal	10005606	7	0	0	0	0
Blood insulin decreased	10005613	23	1	4.3	1	4.3
Blood lactic acid increased	10005635	826	47	5.7	6	0.7
Blood osmolarity increased	10005697	112	16	14.3	3	2.7
Blood triglycerides increased	10005839	5404	1199	22.2	35	0.6
Body mass index decreased	10005895	59	14	23.7	0	0
Body mass index increased	10005897	112	29	25.9	0	0
Central obesity	10065941	81	7	8.6	1	1.2
Coma	10010071	10703	1018	9.5	253	2.4
Dehydration	10012174	27804	1067	3.8	1025	3.7
Depressed level of consciousness	10012373	10200	819	8	333	3.3
Diabetes complicating pregnancy	10012596	3	1	33.3	0	0
Diabetes mellitus	10012601	15780	5523	35	98	0.6
Diabetes mellitus inadequate control	10012607	3689	825	22.4	25	0.7
Diabetes with hyperosmolarity	10012631	27	8	29.6	0	0
Diabetic coma	10012650	1045	551	52.7	1	0.1
Diabetic hepatopathy	10071265	0	0	0	0	0
Diabetic hyperglycaemic coma	10012668	80	7	8.8	1	1.3
Diabetic hyperosmolar coma	10012669	170	66	38.8	7	4.1
Diabetic ketoacidosis	10012671	2725	1090	40	26	1
Diabetic ketoacidotic hyperglycaemic coma	10012672	32	6	18.8	0	0
Fructosamine increased	10017395	5	0	0	0	0
Gestational diabetes	10018209	594	140	23.6	15	2.5
Glucose tolerance decreased	10018428	13	0	0	0	0
Glucose tolerance impaired	10018429	1058	260	24.6	6	0.6
Glucose tolerance impaired in pregnancy	10018430	3	1	33.3	0	0
Glucose tolerance test abnormal	10018433	36	3	8.3	0	0
Glucose urine present	10018478	318	23	7.2	15	4.7
Glycosuria	10018473	384	140	36.5	5	1.3
Glycosuria during pregnancy	10018475	1	0	0	0	0
Glycosylated haemoglobin increased	10018484	2569	171	6.7	11	0.4
Hunger	10020466	1575	142	9	8	0.5
Hypercholesterolaemia	10020603	2210	256	11.6	26	1.2
Hyperglycaemia	10020635	7844	1382	17.6	129	1.6
Hyperglycaemic hyperosmolar nonketotic syndrome	10063554	184	98	53.3	7	3.8
Hyperglycaemic seizure	10071394	5	0	0	0	0
Hyperglycaemic unconsciousness	10071286	10	0	0	0	0
Hyperlactacidaemia	10020660	333	13	3.9	5	1.5
Hyperlipidaemia	10062060	4585	747	16.3	45	1
Hyperosmolar state	10020697	113	24	21.2	3	2.7
Hyperphagia	10020710	632	157	24.8	4	0.6
Hypertriglyceridaemia	10020869	1127	154	13.7	14	1.2
Hypoglycaemia	10020993	10839	672	6.2	99	0.9
Hypoinsulinaemia	10070070	1	0	0	0	0
Impaired fasting glucose	10056997	67	22	32.8	0	0
Impaired insulin secretion	10052341	21	0	0	0	0
Increased appetite	10021654	2646	494	18.7	21	0.8
Increased insulin requirement	10021664	31	2	6.5	0	0
Insulin autoimmune syndrome	10022472	23	0	0	0	0
Insulin resistance	10022489	297	75	25.3	0	0
Insulin resistance syndrome	10022490	18	6	33.3	0	0
Insulin resistant diabetes	10022491	27	8	29.6	0	0
Insulin tolerance test abnormal	10022494	3	0	0	0	0
Insulin-requiring type 2 diabetes mellitus	10053247	122	60	49.2	0	0
Ketoacidosis	10023379	640	250	39.1	3	0.5
Ketonuria	10023388	188	63	33.5	5	2.7
Ketosis	10023391	100	13	13	3	3
Lactic acidosis	10023676	4561	119	2.6	61	1.3
Latent autoimmune diabetes in adults	10066389	16	0	0	0	0
Lipids increased	10024592	368	57	15.5	1	0.3
Loss of consciousness	10024855	28249	1750	6.2	355	1.3
Metabolic acidosis	10027417	5512	253	4.6	121	2.2
Metabolic syndrome	10052066	392	197	50.3	2	0.5
Neonatal diabetes mellitus	10028933	3	0	0	1	33.3
Obesity	10029883	2787	1211	43.5	23	0.8
Overweight	10033307	442	114	25.8	3	0.7
Pancreatogenous diabetes	10033660	6	2	33.3	0	0
Polydipsia	10036067	1026	271	26.4	16	1.6
Polyuria	10036142	1444	197	13.6	27	1.9
Slow response to stimuli	10041045	161	37	23	7	4.3
Thirst	10043458	2595	224	8.6	40	1.5
Type 1 diabetes mellitus	10067584	1252	590	47.1	7	0.6
Type 2 diabetes mellitus	10067585	5272	2862	54.3	16	0.3
Underweight	10048828	111	8	7.2	2	1.8
Unresponsive to stimuli	10045555	5657	442	7.8	123	2.2
Urine ketone body present	10057597	304	31	10.2	13	4.3
Weight decreased	10047895	42275	1765	4.2	466	1.1
Weight increased	10047899	30417	5070	16.7	867	2.9

*With adverse events of interest.

**Figure 1:**
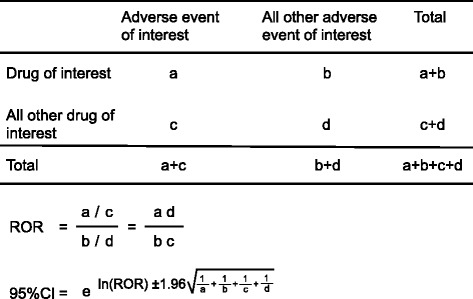
**Two by two contingency table for analysis.**

For signal detection, we calculated the reporting odds ratio (ROR), an established pharmacovigilance index, using a disproportionality analysis. The ROR is calculated as a*d/b*c (Figure 1). The ROR is the ratio of the odds of reporting a specific adverse event versus all other adverse events for a given drug (antipsychotics), compared to the reporting odds for all other drugs present in the database. RORs were expressed as point estimates with 95% confidence intervals (CI). The detection of a signal was dependent on the signal indices exceeding a predefined threshold. Safety signals were considered significant when the ROR estimates and the lower limits of the 95% CI were greater than 2 [16]. We analyzed the effects of monotherapy, two-drug polypharmacy, and three-drug polypharmacy. Data analyses were performed using JMP 9.0 (SAS Institute Inc., Cary, NC, USA).

### Results

The FAERS database contains 4,746,890 reports from January 2004 to March 2013. After excluding duplicates according to the FDA’s recommendation and extracting the reports with complete age and gender information, 2,257,902 reports were analyzed. Using the SMQ “hyperglycemia/new onset diabetes mellitus” (SMQ20000041), we identified 241,478 adverse hyperglycemic events. The reporting ratios and RORs (95% CI) for adverse hyperglycemic events are summarized in Table 1. The reporting ratios of adverse hyperglycemic events in AAPs and typical antipsychotics (TAPs) were 21.8% (21151/96841) and 20.2% (3948/19569), respectively. The number of adverse hyperglycemic events among the top eight reported drugs, quetiapine, olanzapine, risperidone, aripiprazole, haloperidol, clozapine, prochlorperazine, and chlorpromazine, was 12,471 (28.9%), 8,423 (37.9%), 5,968 (27.0%), 4,045 (23.7%), 3,445 (31.5%), 2,614 (14.3%), 1,800 (19.8%), and 1,003 (35.7%), respectively. Each reporting ratio and ROR was analyzed based on administration (monotherapy, two-drug combination, and three-drug combination; Table 4). The RORs (95% CI) for monotherapy with quetiapine, olanzapine, risperidone, aripiprazole, haloperidol, clozapine, prochlorperazine, and chlorpromazine were 2.3 (95% CI: 2.3-2.4), 3.7 (95% CI: 3.6-3.8), 1.5 (95% CI: 1.5-1.6), 1.4 (95% CI: 1.3-1.5), 2.8 (95% CI: 2.7-3.0), 1.1 (95% CI: 1.0-1.1), 2.0 (95% CI: 1.9-2.1), and 1.6 (95% CI: 1.3-1.8), respectively. In contrast, the RORs (95% CI) for three-drug combination therapy were 16.5 (95% CI: 15.1-18.0), 12.0 (95% CI: 11.0-13.2), 12.0 (95% CI: 10.9-13.1), 10.3 (95%: CI 9.1-11.6), 5.9 (95% CI: 5.3-6.7), 2.3 (95% CI: 2.0-2.8), 6.0 (95% CI: 3.6-10.0), and 5.6 (95% CI: 4.5-6.9), respectively.

**Table 4:** **Reporting ratio and ROR for antipsychotic polypharmacy**

	**Drugs** ^*^	**Total**	**Cases** ^**^	**Reporting ratio (%)**	**ROR (95%CI)**
*Atypical*					
	Aripiprazole				
	mono	11457	1645	14.4	1.4(1.3-1.5)
	two	3499	927	26.5	3.0(2.8-3.3)
	three	1099	606	55.1	10.3(9.1-11.6)
	Clozapine				
	mono	13466	1515	11.3	1.1(1.0-1.1)
	two	3486	584	16.8	1.7(1.5-1.8)
	three	750	164	21.9	2.3(2.0-2.8)
	Olanzapine				
	mono	13935	4226	30.3	3.7(3.6-3.8)
	two	4862	1908	39.2	5.4(5.1-5.8)
	three	1904	1121	58.9	12.0(11.0-13.2)
	Quetiapine				
	mono	32942	7114	21.6	2.3(2.3-2.4)
	two	6413	2556	39.9	5.6(5.3-5.9)
	three	2175	1441	66.3	16.5(15.1-18.0)
	Risperidone				
	mono	13820	2154	15.6	1.5(1.5-1.6)
	two	4860	1476	30.4	3.7(3.4-3.9)
	three	1917	1128	58.8	12.0(10.9-13.1)
*Typical*					
	Chlorpromazine				
	mono	1117	175	15.7	1.6(1.3-1.8)
	two	724	179	24.7	2.7(2.3-3.2)
	three	355	142	40.0	5.6(4.5-6.9)
	Haloperidol				
	mono	5604	1420	25.3	2.8(2.7-3.0)
	two	3102	704	22.7	2.5(2.3-2.7)
	three	1079	448	41.5	5.9(5.3-6.7)
	Prochlorperazine				
	mono	8514	1634	19.2	2.0(1.9-2.1)
	two	487	111	22.8	2.5(2.0-3.0)
	three	62	26	41.9	6.0(3.6-10.0)

*Monotherapy and polypharmacy of each antipsychotic.

**With adverse events of interest.

### Discussion

Our results suggest that several antipsychotics increase adverse hyperglycemic events, and that antipsychotic polypharmacy may influence these events using the FAERS database.

In a previous cohort study, olanzapine and clozapine were associated with increased risk for type 2 diabetes [1,2,17]. Citrome *et al.* suggested that exposure to multiple AAPs significantly increased the risk of treatment-emergent diabetes mellitus, as compared to TAPs [1]. However, they discussed that their study design does not permit the quantification of differences between AAPs and the risk of emergent diabetes [1]. Another research group reported that AAP administration results in a small increase, as compared to TAP administration [18]. In our study, the reporting ratio of adverse hyperglycemic events in AAPs (21.8% [21151/96841]) and TAPs (20.2% [3948/19569]) were similar. Thus, we could not obtain meaningful results regarding the difference between AAP administration and TAP administration using the reporting ratio of hyperglycemic adverse events.

The lower limits of the ROR 95% CI for olanzapine, quetiapine, and haloperidol monotherapy were greater than 2 (Table 4). Baker *et al*. reported that olanzapine (AAP), clozapine (AAP), and risperidone (AAP) were associated with hyperglycemic adverse events, whereas aripiprazole (AAP), haloperidol (TAP), and ziprasidone (AAP) had a low association in the FAERS database. We do not have a conclusive explanation for the differences in reporting ratio between the previous report [19] and our findings. One plausible reason could be differences in the terms selected for adverse hyperglycemic events in the MedDRA database. Our study used 93 PTs, whereas Baker *et al.* used 24. Additionally, different datasets were used for the analyses. Baker *et al.* performed their analysis using cumulative subsets from 1968 to 2006, whereas our group utilized datasets from 2004 to 2013.

In this study, each reporting ratio and ROR increased with increasing number of drugs administered (Table 4). The ROR of the three-drug polypharmacy had the highest value for every antipsychotic. Therefore, antipsychotic-induced adverse hyperglycemic events may be influenced by the number of drugs administered. However, the lower limit of the clozapine ROR 95% CI was less than 2. Since the administration of clozapine is not recommended as a first-line treatment [20], physicians may be unlikely to use clozapine in diabetic patients. Therefore, the signal for adverse hyperglycemic events following clozapine might be not detected. Antipsychotic monotherapy and polypharmacy to treat schizophrenia and bipolar disorder has been compared to understand its risk-benefit profile [11,14]. In general, polypharmacy using antipsychotics is not recommended [7-9]. Baker *et al.* evaluated the adverse events signals for each AAP. However, they did not evaluate the effect of antipsychotic polypharmacy on hyperglycemia. Our results suggest that antipsychotic polypharmacy may influence adverse hyperglycemic events. Therefore, clinician should comply with guidelines [7-10] and monitor for adverse polypharmacy-induced hyperglycemic events.

The mechanism by which antipsychotics induce adverse hyperglycemic events remains unclear. AAPs are associated with clinically significant weight gain, and have raised significant concerns regarding possible association with hyperglycemia and type 2 diabetes [1,11,18,19]. Obesity or diabetes may be confounders for adverse hyperglycemic events. However, detailed information, including patient background and diagnosis, is not included in the FAERS database. Therefore, it is difficult to define and stratify the patients investigated.

The FAERS database is subject to various biases, including the exclusion of healthy individuals, the lack of denominator, and confounding factors [21]. Because of these deficits within the spontaneous reporting, ROR do not allow for risk quantification. Rather, the RORs offer a rough indication of the signal strength [21]. Therefore, special attention has to be paid to the interpretation of results from the FAERS database. Other epidemiological studies are required to determine the true risk of adverse hyperglycemic events.

Despite the limitations inherent to spontanesous reporting, we obtained reasonable results in the context of the reported literature. The reporting ratio and ROR suggested an association between antipsychotic drugs and hyperglycemic adverse events, and the reporting ratio was increased with an increase in the number of co-administered antipsychotic drugs. Our study indicates the importance of comparing drug safety profiles using post-marketing real-world data. This information could be useful to improve schizophrenia and bipolar disorder management.

## Abbreviations

FDA: The Food and Drug Administration
AAP: Atypical antipsychotic
FAERS: The FDA adverse event reporting system
ICH: The International Conference on Harmonization
MedDRA: The medical dictionary for regulatory activities
SMQ: The Standardized MedDRA queries
PT: Preferred terms
ROR: Reporting odds ratio
CI: Confidence intervals
TAP: Typical antipsychotic
